# Chronic Exposure to Static Magnetic Fields from Magnetic Resonance Imaging Devices Deserves Screening for Osteoporosis and Vitamin D Levels: A Rat Model

**DOI:** 10.3390/ijerph120808919

**Published:** 2015-07-30

**Authors:** Harun R. Gungor, Semih Akkaya, Nusret Ok, Aygun Yorukoglu, Cagdas Yorukoglu, Esat Kiter, Emin O. Oguz, Nazan Keskin, Gulcin A. Mete

**Affiliations:** 1 Orthopedics and Traumatology Department, Pamukkale University Medical Faculty, Denizli 20070, Turkey; E-Mails: semihakkaya@yahoo.com (S.A.); oknusret@gmail.com (N.O.); cagdasyorukoglu@hotmail.com (C.Y.); esatkiter@gmail.com (E.K.); 2 Pathology Department, Servergazi State Hospital, Denizli 20100, Turkey; E-Mail: cerkezay@hotmail.com; 3 Histology and Embriology Department, Pamukkale University Medical Faculty, Denizli 20070, Turkey; E-Mails: oguzemin@yahoo.com (E.O.O.); nkeskin@pau.edu.tr (N.K.); gabban@pau.edu.tr (G.A.M.)

**Keywords:** Static magnetic field, magnetic resonance imaging, chronic exposure, vitamin D, osteoporosis, bone

## Abstract

Technicians often receive chronic magnetic exposures from magnetic resonance imaging (MRI) devices, mainly due to static magnetic fields (SMFs). Here, we ascertain the biological effects of chronic exposure to SMFs from MRI devices on the bone quality using rats exposed to SMFs in MRI examining rooms. Eighteen Wistar albino male rats were randomly assigned to SMF exposure (A), sham (B), and control (C) groups. Group A rats were positioned within 50 centimeters of the bore of the magnet of 1.5 T MRI machine during the nighttime for 8 weeks. We collected blood samples for biochemical analysis, and bone tissue samples for electron microscopic and histological analysis. The mean vitamin D level in Group A was lower than in the other groups (*p* = 0.002). The mean cortical thickness, the mean trabecular wall thickness, and number of trabeculae per 1 mm^2^ were significantly lower in Group A (*p* = 0.003). TUNEL assay revealed that apoptosis of osteocytes were significantly greater in Group A than the other groups (*p* = 0.005). The effect of SMFs in chronic exposure is related to movement within the magnetic field that induces low-frequency fields within the tissues. These fields can exceed the exposure limits necessary to deteriorate bone microstructure and vitamin D metabolism.

## 1. Introduction

Magnetic resonance imaging (MRI) devices produce three types of magnetic fields during image acquisition, including static magnetic fields (SMFs) that produce magnetization vectors in the body, a gradient magnetic field for aligning protons inside the body, and radio-frequency electromagnetic waves for detecting magnetization vectors by the MRI scanner [1,2,3,4,5]. Although all of these magnetic fields are employed during an MRI examination, technicians are mainly exposed to SMFs since they stand outside the examining room while the scanning is underway. This exposure occurs during positioning of the patient and coil selection in vicinity of the magnet when the MRI machine is idle [1,2,3,4]. Static field surveys show that fields of 200 mT exist approximately 50 centimeters from the bore opening of the magnet for most 1.5 T MRI systems [3,4]. In addition, the movement of a person within a SMF induces low-frequency fields in tissues that may exceed expected exposure limits [4].

There have been many studies focusing on the biological effects of chronic exposure to SMFs but research related to the health effects of chronic exposure to SMFs on bone is scarce. The majority of studies about the effects of SMFs on the skeleton is at the cellular level or is applied to animal models [6,7]. Almost all evidence from these studies suggests either beneficial effects or no harmful bio-effects of SMFs on bone health [6,7,8,9,10,11,12,13,14,15,16,17,18,19,20,21,22]. However, most of these animal studies in rodents and research at the cellular level evaluating the effects of SMFs on the skeletal system have often been complicated by creating pure SMFs *in vivo* and simulating this chronic exposure due to relative movement between the magnet and bone in real life. In reality, the effect of SMFs is not a simple stimulation that supplies any form of energy to the body, but rather the movement of a person within an SMF will induce fields within the tissues [4]. Therefore, these cellular studies and rodent experiments up until now have not accurately represented information about the effects of chronic exposure to SMFs on bones.

In this study, we ascertain the biological effects of chronic exposure to SMFs from MRI devices on the bone quality and bone turnover blood biochemistry markers using rats exposed to SMFs in MRI examining rooms.

## 2. Experimental Section

### 2.1. Animals and Exposure Procedure

Eighteen male Wistar albino rats (230 ± 15 g body weight and approximately 20 weeks old) were used in the experiments. The rats were randomly selected and housed in polycarbonate cages that were wide enough for the rats to move freely (50 × 75 cm) with free access to tap water and rat chow with a dark/light cycle of 12:12 h. The temperature was 21 ± 2 °C and the relative humidity was 50%–70%. The experiment was designed by performing power analysis to obtain reliable data with a minimum number of animals. The rats were randomly assigned into SMF exposure (Group A; *n* = 6), sham (Group B; *n* = 6), and control (Group C; *n* = 6) groups. The rats in the Groups A and B were brought from the laboratory to the MRI facility in polycarbonate cages at midnight, when the 1.5 T MRI machine (GE Signa Excite HD; GE Medical Systems, Milwaukee, WI, USA) was in a stationary mode; this procedure was repeated every day for 8 weeks. The Group A rats were placed within 50 cm of the bore of the magnet. The Group B rats were placed in the technician’s room, where they are isolated from the SMF. Both group of rats stayed at the MRI facility during the night until 6 am, and then they were returned to the laboratory. The night period for SMF exposure was purposely chosen since rodents are nocturnal animals. The Group C rats were kept in animal laboratories during the entire period of the study. At the end of the 8 weeks of observations, all of the rats were anesthetized via an intra-peritoneal injection of xylazine (10 mg/kg) and ketamine (90 mg/kg) combination and sacrificed by exsanguinations. Intra-cardiac blood samples for biochemistry analysis, bone tissue samples from the right femoral sub-capital region for electron microscopic analysis, and bone tissue samples from the left femoral sub-capital region for histological analysis were collected. The study was approved by Pamukkale University Medical Faculty Institutional Review for Animal Research Board and conducted in accordance with institutional guidelines (PAUHDEK-2013/033).

### 2.2. Biochemistry Analysis

Commercial ELISA kits were employed for quantifying serum biochemical markers for vitamin D (Rat Vitamin D, VD ELISA Kit, CusoBio^®^, Hubei, China), and bone-specific alkaline Phosphatase (Rat bone alkaline phosphatase, BALP ELISA Kit, CusoBio^®^, Hubei, China). Assays were performed according to the manufacturer’s instructions. Calcium and phosphorus levels were analyzed by spectrophotometric methods in an auto-analyzer (Roche Cobas 8000, Roche-Hitachi Diagnostics, Tokyo, Japan).

### 2.3. Electron Microscopy and FESEM Characterization

**Figure 1:**
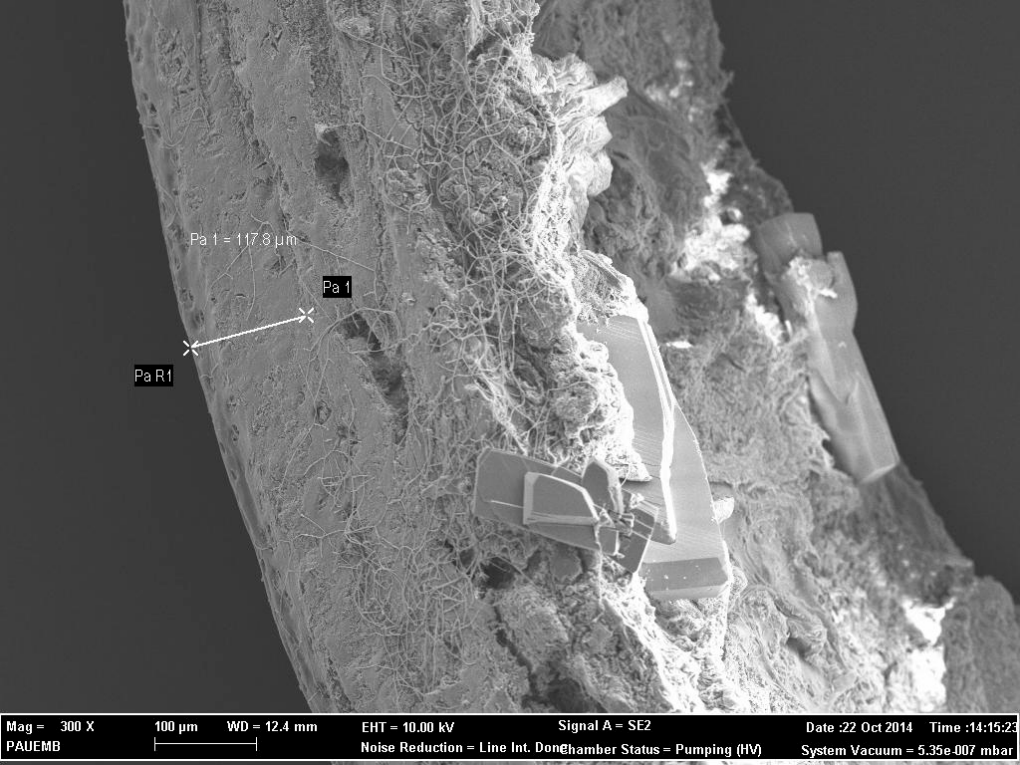
Measurement of cortical bone thickness (at 300× magnification) on the FESEM image (specimen from Group A).

The bone samples from the right femur beginning from sub-capital region towards the neck were fixed in glutaraldehyde solution for scanning electron microscopy. After being washed in sodium phosphate buffered solution, the samples were dehydrated in acetone at gradually increasing concentrations, then critical point-dried, mounted on stubs, and sputter-coated with gold. The observations were carried out with a field emission electron microscope (FESEM; Carl Zeiss, Supra 40 VP, Oberkochen, Germany). Cortical bone thicknesses (at 300× magnification) for each specimen were measured on the FESEM images using software system of the FESEM. Borders of cortices were marked on real time images and the software calculated the distance between these makers (Figure 1). Three successive measurements were performed at three different sites of the cortex of each specimen and average of the measurements was recorded as the cortical thickness of specific specimen to determine the quality of the femoral neck bone architecture [23,24].

### 2.4. Transmission Electron Microscopy

For transmission electron microscopy (TEM), fragments of the right femur beginning from sub-capital region towards the neck were fixed in glutaraldehyde solution in PBS and then decalcified with 4% EDTA for 20 days. The samples were postfixed in 1% osmium in PBS, dehydrated in graded ethanols, cleared in toluene, and embedded in Epon 812. Ultrathin sections were stained with uranyl acetate and lead citrate, and examined using a STEM detector in a FESEM (CarlZeiss, Supra 40 VP, Oberkochen, Germany).

### 2.5. Histological Evaluation

Specimens from the left femur beginning from sub-capital region towards the neck of rats were fixed with 10% neutral formalin and decalcified with 5% nitric acid solution for 1 day. For light microscopy, the routine bone-tissue processing was performed and tissues were embedded in paraffin. Cross sections were taken in 4-mm intervals and stained with hematoxylin and eosin for evaluation of trabecular bone quality at 4× and 10× magnifications in light microscopy.

### 2.6. Histomorphometric Evaluation for Trabecular Thickness and Trabeculae Number

**Figure 2:**
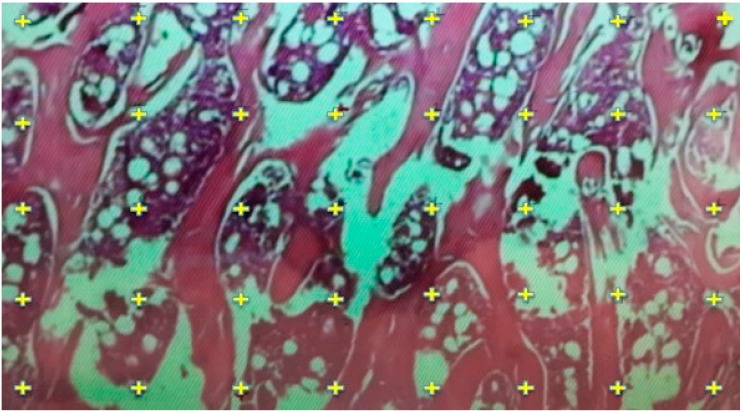
Measurement of trabecular bone thickness and determination of number of trabeculae (on a chart area of approximately 878 × 1250 µm^2^ at 4× magnification) on the histologic specimen (specimen from Group A).

Number of trabeculae in each cross section of histogical specimen counted on a chart area of approximately 878 × 1250 µm^2^ at 4× magnification (Figure 2). The average number of trabeculae per 1 mm^2^ area was calculated for each rat (number of trabeculae in 1 mm^2^ / number of cross sectional specimen for each rat). Thickness of each trabeculae was measured in designated area of each slice of the specimen using the software of the computer that the measurements were done, and average thickness of the measured trabecular width was assigned for evaluation of the specific specimen (sum of the thickness of the trabeculae on each slice of specimen / total number of trabeculae on each slice of specimen).

### 2.7. TUNEL Analysis for Apoptosis Measurements

Apoptotic cells were determined using the transferase mediated deoxyuridine triphosphate (dUTP)-digoxigenin nick-end labeling (TUNEL) assay (In Situ Cell Death Detection Kit, Roche, Mannheim, Germany) applied to the paraffin sections. The sections were stained with methyl green solution and analyzed by using a light microscope. For quantitative measurement of the number of cells that underwent apoptosis, 100 cells were randomly counted in cells showing apoptotic morphology in the bone, and apoptotic cell percentages were calculated.

### 2.8. Statistical Analysis

The data were analyzed using Statistical Package for Social Sciences software (SPSS Version 17, Chicago, IL, USA). Descriptive statistics (including mean ± standard deviation, frequency and percentage) were calculated for Groups A, B, and C, and for the entire cohort of rats. For parametric and non-parametric tests, the difference between the means of variables in groups was compared using an independent samples *t*-test and a Mann-Whitney U-test, respectively. A chi-squared test was used to compare categorical variables. Pearson's correlation coefficient was used to determine whether there is a correlation between continuous variables. The statistical significance was set at *p* < 0.05.

## 3. Results

The results are summarized in Table 1.

**Table 1:** Mean values and standard deviations of measured variables.

Measured Variables	Group A	Group B	Group C	*p* Value
	(Mean ± SD)	(Mean ± SD)	(Mean ± SD)	
	(Min–Max)	(Min–Max)	(Min–Max)	
Vitamin D (pg/mL)	38.7 ± 20.2	97.5 ± 10.2	82.2 ± 13.8	0.002
	(17.4–73.2)	(78.6–109.7)	(64.6–95.7)	
A. phosphatase (mIU/mL)	1 ± 0.97	0.9 ± 0.2	0.8 ± 0.2	n.s
	(0.12–3.11)	(0.4–1.5)	(0.5–1)	
Calcium ( mg/dL)	8.9 ± 0.7	9 ± 0.2	9 ± 0.7	n.s
	(7.8–9.7)	(8.7–9.1)	(7.8–9.9)	
Phosphorus (mg/dL)	7.4 ± 0.8	7.1 ± 0.6	7.3 ± 1.3	n.s
	(6.4–8.5)	(6.3–7.7)	(6.1–9.6)	
Cortical thickness (µm)	156.9 ± 30.4	254.9 ± 27.7	293.7 ± 26.6	0.001
	(103.6–186.8)	(224–283.8)	(262.8–327.2)	
Trabecular thickness (µm)	39.7 ± 4.1	60.1 ± 8.7	61 ± 8.3	0.003
	(10.6–22.9)	(51.1–71.5)	(50.2–72.4)	
Trabeculae n/mm^2^	4.4 ± 0.5	5.6 ± 0.3	5.6 ± 0.4	0.003
	(3.7–4.9)	(5.3–6)	(5.2–6.1)	
Apoptotosis (%)	41.1 ± 2.8	26.4 ± 4.7	4.1 ± 1.8	0.005
	(37.8–44.1)	(20.3–32.1)	(1.8–6.1)	

### 3.1. Biochemistry Analysis

In biochemistry analyses, the mean vitamin D level in Group A was lower than in the other groups. There was a statistically significant difference between Group A rats and rats in the other groups (*p* = 0.002). The mean values for rat bone alkaline phosphatase, calcium, and phosphorus were similar for all groups with no statistically significant differences.

### 3.2. Electron Microscopy and FESEM Results

In FESEM measurements, the mean cortical thickness was significantly lower in Group A than in the other groups (*p* = 0.001).

### 3.3. Transmission Electron Microscopy Results

**Figure 3:**
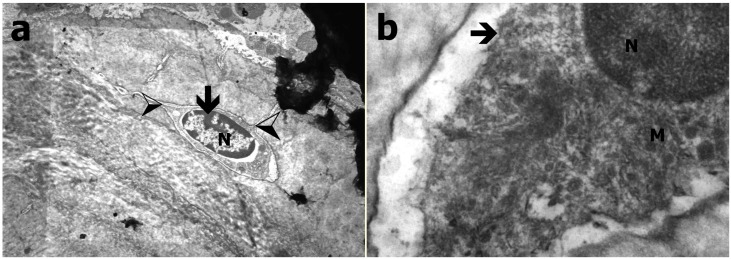
TEM evaluation of Group C; (**a**) Osteocyte within lacunae (black arrow) and cytoplasmic extensions (black and white arrow), N refers to nucleus (at 15K×); (**b**) Osteocyte (black arrow), M refers to mitochondria (at 135K×).

**Figure 4:**
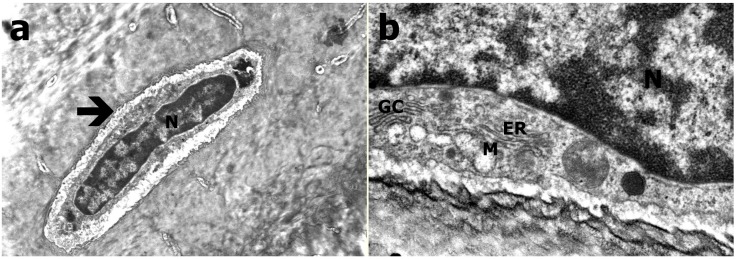
TEM evaluation of Group B (**a**) Osteocyte (arrow; N refers to nucleus; at 21K×); (**b**) Granular endoplasmic reticulum (ER), mitochondria (M), golgi complex (GC; at 83K×).

**Figure 5:**
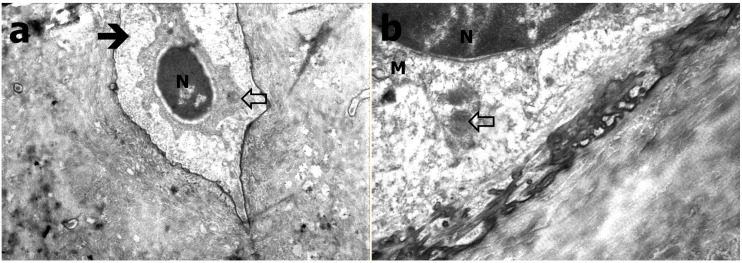
TEM evaluation of Group A (**a**) Heterochromatic osteocyte (black arrow), decreased and condensed cytoplasm with round appearances (white arrow; at 26K×); (**b**) Round appearances within cytoplasm (white arrow), mitochondria (M) and nucleus (N; at 56K×).

In TEM evaluation, the osteocytes were settled in bone lacunas. The cytoplasmic extensions protruding into the neighboring cells were evident in Group C, and a large number of round mitochondria were observed in cytoplasm. The chromatin distribution in the nucleus was also in adequate form in Group C (Figure 3). In Group B, it was observed that the osteocytes were narrowed compared to those in the control group. The cumulation in amorphous fashion in cytoplasm was distinctive. There was degeneration and crystallization in some mitochondria. GER and Golgi were in their normal appearance. An increase in heterochromatic areas of the nucleus was observed (Figure 4). The osteocytes in Group A were smaller than those in the control group. The observed cytoplasm was decreased, and the cytoplasmic extensions to the neighboring cells were disappeared in Group A. Interestingly, the structures in round appearances that are most probably mitochondria were observed in largely disordered and scattered cytoplasm. It was also observed that the chromatin distribution in the nucleus was changed and had a heterochromatic appearance (Figure 5).

### 3.4. Results of Histological Evaluation

**Figure 6:**
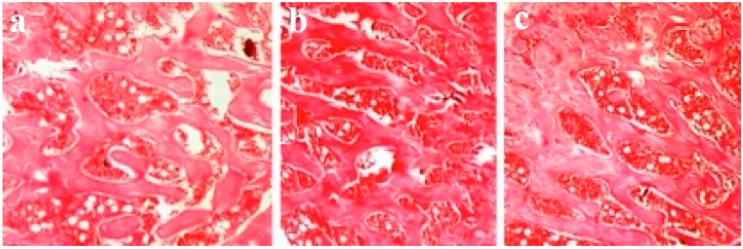
Hematoxylin and eosin staining of bone sections of left femur subcapital region; (**a**) In Group A, trabecular walls are thinner than in other groups (**b**) Group B; (**c**) Group C (10×).

In histological specimens, tissues of Group B and C rats revealed uniform bone structure. However, in specimens from Group A rats, there were irregularities and thinning in the trabecular walls along with adjacent regeneration areas, and osteoporosis characterized by increased adipose tissue and vascularity in the inter-trabecular distance (Figure 6).

### 3.5. Results of Histomorphometric Evaluation for Trabecular Thickness and Trabeculae Number

In histomorphometric evaluation, both trabecular thickness and number of trabeculae per 1 mm^2^ in Group A rats were found to be less than the other groups (*p* = 0.003, *p* = 0.003, respectively).

### 3.6. Results of TUNEL Analysis for Apoptosis Measurements

**Figure 7:**
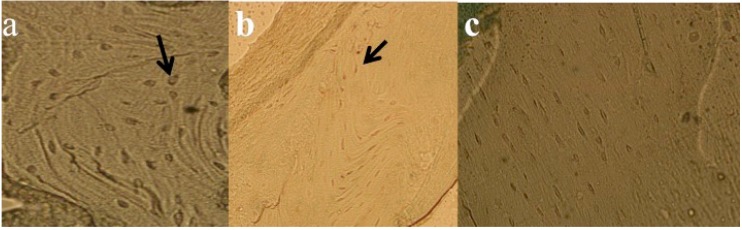
Tunnel assay, arrows point apoptotic cells (**a**) Group A; (**b**) Group B; (**c**) Group C (at 20×).

TUNEL assay revealed that mean percentage of apoptotic osteocytes was significantly greater in SMF exposed rats than sham and control group (*p* = 0.005; Figure 7).

### 3.7. Correlations

There was no correlation between the measured biochemical and electron microscopic parameters within the assigned groups except there was a significant correlation between cortical thickness and number of trabeculae per 1mm^2^ (*p* = 0.005, *r* = 0.943), and between the number of trabeculae per 1 mm^2^ and calcium levels in Group A (*p* = 0.042, *r* = −0.829). This means that as the cortical thickness increases the number of trabeculae per mm2 increases and as the number of trabeculae per 1 mm^2^ increases the calcium levels decreases in contrast to other groups in Group A.

## 4. Discussion

The most important finding of this study is the negative effects of chronic exposure to SMFs from MRI devices on bone health. Chronic exposure of the rats—freely moving in their cages, just like the occupational exposure of staff from MRI magnets—resulted in the deterioration of femoral neck bone microstructure and a reduction in vitamin D levels.

The majority of cellular studies about the effects of SMFs on bone tissue are related to osteoblasts. Yang *et al.*, [25] studied human osteoblast-like cells. These authors placed culture dishes directly on the surface of a permanent magnet with a flux density of 0.4 T and found a 1.24-fold increase in alkaline phosphatase activity by calmodulin signaling pathway at 3 days. Huang *et al.*, [26] studied specific osteoblastic cell lines exposed to a 0.4 T SMF. They found promotion of the cells only when the cells were seeded at a specific initial cellular density. This study revealed that moderate SMFs promoted the differentiation of specific osteoblast cell lines at 1–3 days with increased expression of alkaline phosphatase, osteoprotegerin, and collagen type I. Cellular studies on osteoclasts have been very few due to some difficulties in isolation of cells. Di *et al.*, [27] studied the human pre-osteoclast cells FLG29.1 and found no difference in the proliferation of cells exposed to SMFs generated by a 16 T superconducting magnet and the proliferation of cells in a control group. However, they also found that the number of differentiated osteoclasts, and the activity of tartrate resistant acid phosphatase were decreased. The osteoclast-inhibiting effects in this study and the osteoblast differentiation-promoting effects in the studies by Yang *et al.*, [25] and Di *et al.*, [27] and in all other similar studies, were attributed to relatively stationary SMFs.

The vast majority of experiments pertaining to the effects of SMFs on bone healing favor-promoting effects of SMFs. Aydin and Bezer [28] implanted a magnetized intramedullary device directly into a fractured rabbit femur and studied the results. They concluded that local SMFs had beneficial effects on bone healing. Xu *et al.*, [29] examined the effects of SMFs in rats with an ischemic femur model. The group of rats that had magnetized rods implanted within their ischemic femurs showed an enhancement in femoral bone formation relative to the control group. In these two bone-healing models, the effects were not the result of a gradient effect on bone tissues.

As in these studies on bone tissues, most cellular experiments and studies of animal models showing either osteogenic or antiresorbtive effects of SMFs are not simulating the effects of chronic exposure to SMFs on bone but rather revealing relatively stationary effects of nearby SMFs [6,7,8,9,10,11,12,13,14,15,16,17,18,19,20,21,22,25,26,27,28,29]. Recently, Aïda *et al.*, [30] exposed rats in plexiglass cages to SMFs of 128 mT for 1 h per day for 5 consecutive days. Their model was imitating exposure from MRI devices, as our study does, with relative movement between the rat and the magnet inducing low-frequency fields in tissues. In the Aïda *et al.*, [30] study, rats exposed to SMFs were depleted in vitamin D. In addition, supplementation of vitamin D during or following SMF exposure either corrected or prevented the vitamin D deficiency.

Vitamin D is essential for musculoskeletal health since it is both involved in calcium absorption from the bowel and mineralization of newly formed osteoid tissue in bone [31,32]. In humans, the most important compounds in vitamin D group are vitamin D3 cholecalciferol (vitamin D_3_) and ergocalciferol (vitamin D_2_) [31]. Cholecalciferol and ergocalciferol can be ingested from the diet and from supplements [31,32]. In addition, dermal synthesis of vitamin D from cholesterol is dependent on sun exposure. In our study, 12 h night and 12 h day light period maintained during whole period of exposure to SMF. To maintain proper feeding of the whole group of the animals, free access to tap water and rat chow was available. Vitamin D levels of sham group were similar to control group in our measurements. Vitamin D either from the diet or dermal synthesis is biologically inactive. Enzymatic conversion of vitamin D (hydroxylation) in the liver and kidney is required for production of active metabolite. In the liver, cholecalciferol (vitamin D_3_) is converted to calcidiol. Ergocalciferol (vitamin D_2_) is converted in the liver to 25-hydroxyergocalciferol. Part of the calcidiol is converted by the kidneys to calcitriol, the biologically active form of vitamin D [33]. Calcitriol circulates as a hormone in the blood and mediates its biological effects by binding to the vitamin D receptor, which is principally located in the nuclei of target cells [34,35]. It maintains skeletal calcium balance by promoting calcium absorption in the intestines, promoting bone resorption by increasing osteoclast number, maintaining calcium and phosphate levels for bone formation, and allowing proper functioning of parathyroid hormone to maintain serum calcium levels. Vitamin D is also critical for bone remodeling through its role as a potent stimulator of bone resorption, although it may initially appear paradoxical [36]. Therefore, Vitamin D deficiency can result in osteoporosis or increased bone fracture risk because a lack of vitamin D alters mineral metabolism in the body [36]. In our study, alkaline phosphatase levels of all groups were similar. This shows that although the trabecular number per mm^2^ and trabecular thickness were decreased in Group A rats, osteoblastic activity were similar in whole groups of rats. We could not measure the parathyroid hormone levels that shows interaction with vitamin D levels, and the parameters showing increased osteoclastic activity such as the C-terminal telopeptide; this is because the amount of blood we collected from the rats were limited (approximately 3-4 cc), and in addition expense of these laboratory tests was beyond the funds of this study. However, the message from the results of this preliminary study is clear that the exposure of the animals to SMF resulted in a decrease in Vitamin D levels along with a decrease in trabecular number per mm^2^ and trabecular thickness although the mechanism resulting in this situation needs to be explained by future studies.

In a previous study by our group [37], adverse effects of SMFs from MRI devices on the bone health of workers were detected. Volunteer MRI technicians working with 1.5 T MRI units were included in the study. The staff exhibited decreased bone mineral density and bone mineral concentrations, along with decreased vitamin D levels, relative to the control group. Results obtained from the current experimental study confirm the findings of our previous study, which is important from the point of view of workers chronically exposed to SMFs. However, symptoms of vitamin D deficiency are unfortunately vague and it can be difficult to ascertain whether a low serum vitamin D level is a causal marker for poor nutrition, or a lack of outdoor activity, or inadequate sun exposure. Nonetheless, according to results of our study, chronic exposure to SMFs is a risk factor that predisposes lower levels of vitamin D. If workers are suspected of having symptoms caused by osteomalacia, or have chronic widespread pain, a decision can be made to measure serum vitamin D as part of their clinical and laboratory evaluation [38,39]. In case that they are asymptomatic, the findings of the both studies suggest that persons who are chronically exposed to SMFs should be tested for osteoporosis and vitamin D depletion. In addition, measures should be taken to prevent and manage osteoporosis, and vitamin D supplementation should be strongly considered for people chronically exposed to SMFs, at least until the interaction between SMFs and bone tissue can be explained.

We could not measure the SMF exposure dosages of rats during our study. The results would be more accurate if we could have done these measurements. Static field studies show that 200 mT is exceeded approximately 50 centimeters from the bore opening of the magnet in 1.5 T MRI devices [3,4]. In addition, bone quality in sham group was also lower in sham group (Group B) in contrast to control group (Group B). However this decrease is much less than as it is in study group (Group A). This may be attributed either to stress riser factors for rats, since they traveled from laboratory to MRI facility during the study, or to insufficient isolation of the technician room from the bore of the magnet. To decide about this problem we should have measured SMFs in these areas.

## 5. Conclusions

The effect of SMFs in terms of chronic exposure is not a simple process that supplies energy to a body. Instead, the movement of a person or animal within a SMF induces low-frequency fields within bodily tissues. These fields can exceed expected exposure limits and deteriorate bone microstructure and vitamin D metabolism. Therefore, additional studies should be planned at the cellular levels and using animal models to explain how SMFs with induced low-frequency fields affect bone metabolism.
